# Priming crops against biotic and abiotic stresses: MSB as a tool for studying mechanisms

**DOI:** 10.3389/fpls.2014.00642

**Published:** 2014-11-12

**Authors:** Andrés A. Borges, David Jiménez-Arias, Marino Expósito-Rodríguez, Luisa M. Sandalio, José A. Pérez

**Affiliations:** ^1^Departamento de Agrobiología, Instituto de Productos Naturales y Agrobiología – Consejo Superior de Investigaciones Científicas (CSIC)La Laguna, Spain; ^2^Biosciences, College of Life and Environmental Sciences, University of ExeterExeter, UK; ^3^Departamento de Bioquímica, Biología Celular y Molecular de Plantas, Estación Experimental del Zaidín – Consejo Superior de Investigaciones Científicas (CSIC)Granada, Spain; ^4^Área de Genética, Instituto Universitario de Enfermedades Tropicales y Salud Pública, Universidad de La LagunaLa Laguna, Spain

**Keywords:** menadione sodium bisulphite (MSB), priming, priming agents, biotic stress, abiotic stress, reactive oxygen species, ROS-dependent signaling networks

## Introduction

Biotic and abiotic stresses are the main problems affecting agricultural losses. Consequently, understanding the mechanisms underlying plant resistance or tolerance helps us to develop fruitful new agricultural strategies. These will allow us to face the challenges of producing food for a growing human population in a sustainable and environmentally friendly way.

To compensate for their sessile life and face a broad range of biotic and abiotic stresses, plants have evolved a wide range of survival and adaptation strategies. Amongst them, higher plants are capable of inducing some stress “memory,” or “stress imprinting.” Bruce et al. (2007) define stress imprinting as genetic or biochemical modifications induced by a first stress exposure that leads to enhanced resistance to a later stress. This phenomenon also known as “priming” results in a faster and stronger induction of basal resistance mechanisms upon subsequent pathogen attack, or greater tolerance against abiotic stresses (Pastor et al., 2013). Basal resistance by itself is too weak to protect against virulent pathogens, since it constitutes a residual level of resistance after immune suppression by the pathogen through co-evolution (Walters and Heil, 2007; Conrath, 2011). However, Ahmad et al. (2010) proposed that priming-inducing stimuli can provide more effective basal resistance, particularly when an earlier defense response precedes immune suppression by the invading pathogen.

Following perception of microbe-associated molecular patterns (MAMPs), recognition of pathogen-derived effectors or colonization by beneficial microbes, priming can also be induced by treatment with some natural or synthetic compounds or even by wounding (Conrath, 2011). Through priming plants are able to induce responses to a range of biotic and abiotic stresses, providing low-cost protection in relatively high stress-pressure conditions. Despite priming phenomena having been widely described, the molecular mechanisms of defense priming are still unclear. Such techniques are now starting to emerge as a promising alternative for sustainable modern pest management in the field, since some pesticides have been shown to actually exert their known plant health- and yield-increasing effects through priming (Beckers and Conrath, 2007). From an ecological point of view, the benefits of priming are clear: rather than leading to the costly and potentially wasteful activation of defenses, a metabolic state of alert is induced after an initial infection, enabling a rapid intense resistance response to subsequent attacks. Thus, this strategy appears promising for crop protection purposes (Walters and Heil, 2007).

## Reactive oxygen species: key molecules in priming

Reactive oxygen species (ROS) such as hydrogen peroxide, superoxide, and hydroxyl radicals are inherent by-products of aerobic metabolism. ROS have not only the potential to cause oxidative damage by reacting with biomolecules, but it is widely accepted that they also have key roles as signaling molecules that contribute to control of plant development and to the sensing of the external environment (Smirnoff, 2005; del Río and Puppo, 2009).

ROS metabolism includes a complex network that interacts closely with hormonal signaling systems and allows plants a subtle regulation of developmental events as well as biotic and abiotic stress responses. Oxidative stress is the term widely used to define the imbalance between ROS production and scavenging or detoxification (Pastor et al., 2013). Recently, a mechanism to explain the role of ROS in cell signaling has been reported. This model proposes that changes in redox homeostasis generate specific ROS signals or ROS waves that, next to other signals such as hormones and small peptides, can prime neighboring cells to defense (Mittler et al., 2011). The afore-mentioned ROS signal waves are sensed by specific receptors that can transfer the message to activate other networks through phosphorylation cascades using mitogen-activated protein kinases (MPKs) (Colcombet and Hirt, 2008). ROS have been involved in priming events induced by biotic and abiotic stimuli, although the mechanisms are so far not well established. One of the challenges in ROS research is to identify specific ROS receptors and to establish how the cell is able to decode endogenous ROS signals and discriminate between different stimuli giving rise to a very specific defense response. In addition to ROS, nitric oxide (NO) is another key signaling molecule involved in different cellular process (Romero-Puertas et al., 2013). It can induce a priming protective effect against biotic and abiotic factors through a complex network, probably involving ROS by inducing antioxidant systems (Sun and Li, 2013), calcium ions and hormones. This area deserves further research.

## MSB: a novel priming agent

Menadione sodium bisulphite (MSB) is a water-soluble addition compound of vitamin K3, or pro-vitamin K. Menadione, previously thought to be synthetic, has been isolated from fungi and phanerogams (Binder et al., 1989). Moreover, it is a redox-active compound widely used in the study of oxidant stress in plants (Sun et al., 1999), mammals (Shi et al., 1996), fungi (Emri et al., 1999), and bacteria (Mongkolsuk et al., 1998). It is promptly subjected to cell-mediated one-electron reduction, generating superoxide radicals (O^−^_2_) and hydrogen peroxide (H_2_O_2_) (Hassan and Fridovich, 1979). The physiological function of vitamin K in plants is associated directly with its redox properties. Quinones, benzoquinones, and naphthoquinones such as menadione have two major chemical properties that render them reactive in biological systems. They may attract electrons acting as oxidant agent or electrophile, and in turn also donate electrons, acting in this case as reducing agent or nucleophile. The grade to which these properties contribute to overall toxicity is highly dependent on the concentration, and the chemical and cellular exposure conditions (Castro et al., 2007). This property can induce an increased production of ROS in which vitamin K3 (within the group formed by vitamins K1 and K2) seems to be more active in the induction of oxidative stress. It has been proposed that vitamin K3 could be converted once metabolized into vitamin K1, but this has not yet been demonstrated (Manzotti et al., 2008). The most studied of such compounds, vitamin K1 or phylloquinone, has been detected inside thylakoid membranes as an electron carrier and key element within the photosystem I redox chain. A recently published review suggests the role of vitamin K as mobile electron carrier in the transport chain transferring electrons across the plasma membrane, and the possibility that this molecule contributes to the maintenance of a suitable redox state of some important proteins embedded in the plasma membrane with protective functions against stress (Lüthje et al., 2013). Phylloquinone is a metabolite of the shikimate pathway widely used by plants and bacteria but not by animals, for this reason they must obtain some compounds including vitamin K through their diet. The physiological function of vitamin K in plants is directly linked to its redox properties deriving from the presence of a double quinone functional group on the naphthalenic ring. In fact, similarly to many other quinones and naphthoquinones, vitamin K can be reduced and reoxidized cyclically by several substances and enzyme pools (Döring and Lüthje, 1996; Lütthje et al., 1998). Given its hydrophobic nature, menadione can easily cross biological membranes, allowing it to enter organelles and and catalyze superoxide, hydrogen peroxide, and hydroxyl radical production (Hassan and Fridovich, 1979; Lehmann et al., 2012). A recent study in Arabidopsis roots using menadione as oxidant showed that ROS are produced by an electron transport chain via mitochondria and plastids (Lehmann et al., 2012). Furthermore, De Nisi et al. (2006) observed that menadione is capable of increasing the activity of H^+^-ATPase. This enzyme uses energy derived from ATP hydrolysis to pump protons from the cytoplasm to the apoplast, which creates and maintains a negative membrane potential and an acid pH in the extracellular space. This electrochemical gradient can control many aspects of transport through the plasma membrane, such as secondary transport control of cell turgor, stomatal closure (Elmore and Coaker, 2011) or the movement of sucrose and amino acids to the cytoplasm by symport transporters (Morsomme and Boutry, 2000). This latter might be involved in regulating the activity of this H^+^-ATPase of the plasma membrane during the defensive response against pathogens (Elmore and Coaker, 2011).

MSB, first studied as a plant growth regulator (Rama-Rao et al., 1985), has been widely demonstrated to function as plant defense elicitor against several pathogens in a number of different plant species (Borges et al., 2003, 2004, 2009; Liu et al., 2006; Pushpalatha et al., 2007; ShengYi et al., 2007). Changes in gene expression in response to 0.2 mM MSB at different time-points post-treatment, using microarray technology, show that MSB leads to a unique molecular mark by inducing differentially the expression of 158 genes. More up-regulated genes were included in categories such as “response to stress” than the background, and the behavior of these genes in different treatments confirmed their role in response to biotic and abiotic stress (Borges et al., 2009). Different applications of MSB in agriculture have been patented (Borges-Pérez and Fernández-Falcón, 1996; Borges-Rodríguez et al., 2008; Borges-Rodríguez and Borges-Pérez, 2010) and several MSB-based commercial formulations have been marketed.

**Figure 1:**
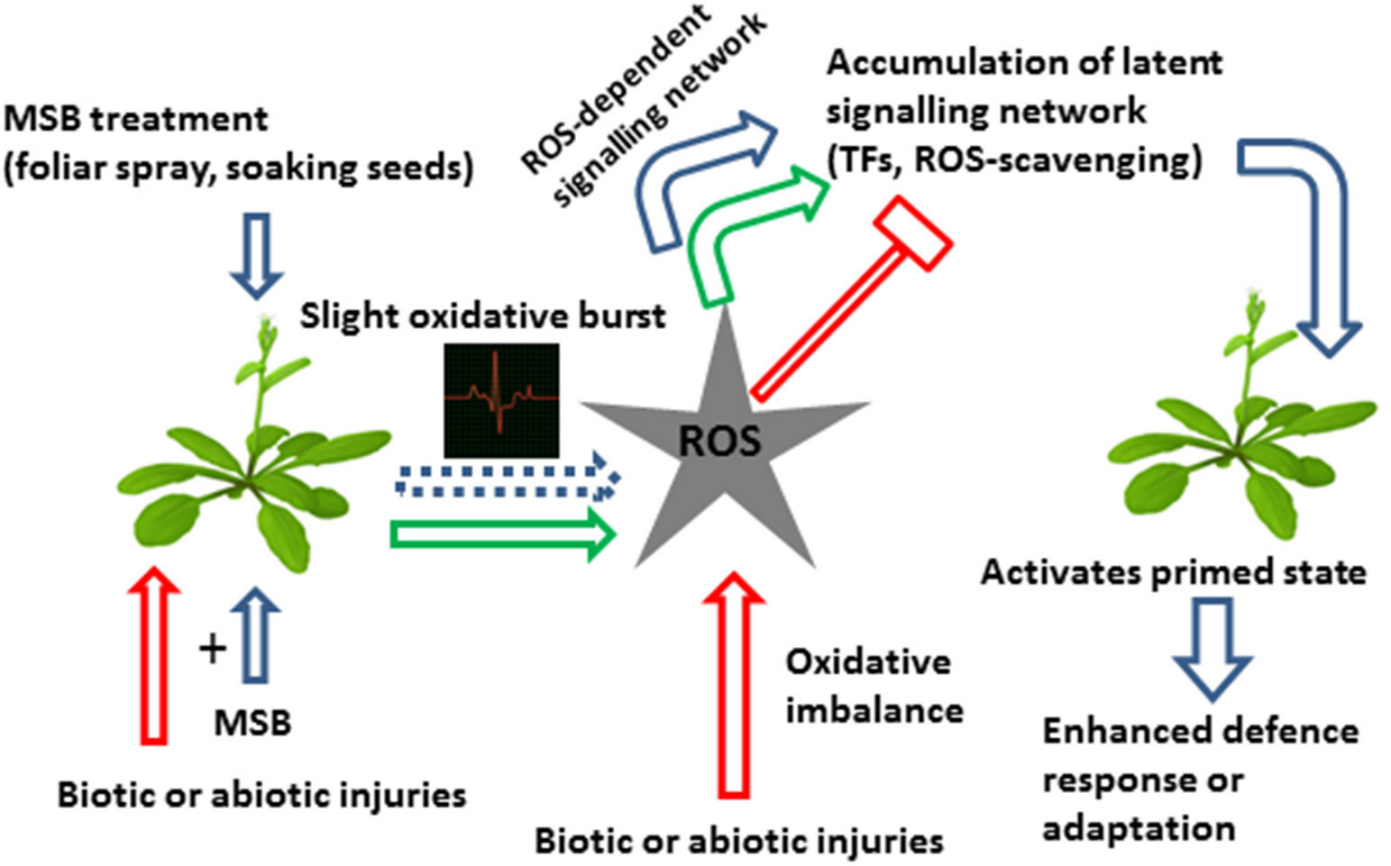
**Hypothetical model on the effects of MSB on plant defense mechanisms against biotic and abiotic stresses**. MSB treatment is capable of inducing resistance by priming through of a slight oxidative burst which develops a ROS-dependent signaling network and inducing the accumulation of latent defense proteins such as ROS-scavenging and transcription factors, among others, resulting in a primed state and an enhanced stress response. Abbreviations: TFs, transcription factors; ROS-scavenging, reactive oxygen species-scavenging.

MSB was capable of inducing resistance by priming in Arabidopsis against the virulent strain *Pseudomonas syringae* pv. tomato DC3000 (Borges et al., 2009). Previous studies in oilseed rape plants (*Brassica napus* cv Bristol) showed that MSB-pretreatment 24 h before inoculation with *Leptophaeria maculans* exhibited rings of necrotic mesophyll cells surrounding the invasive hyphae of *L. maculans*, after staining with aniline blue in lactophenol. In water pre-treated control plants, unobstructed *L. maculans* hyphal growth was observed at infection sites, with no visible host reaction (Liu et al., 2006). However, staining assays of MSB-treated Arabidopsis plants did not fit with the generation of ROS or SAR *in planta*, despite the fact that a significant up-regulation of genes involved in ROS detoxification was found in the microarray. In this interaction, MSB induced resistance by priming without inducing necrosis or visible damage (Borges et al., 2009). Furthermore, a western blot analysis of the known SA signaling pathway marker PR1 (Dong, 2001) showed that MSB does not itself induce PR1 protein expression. Contrastingly, 3 days after inoculation, MSB-pretreated plants enhanced more than two-fold PR1 expression as compared with mock plants (Borges et al., 2009). Finally, the promoter analysis of MSB-induced *cis*-elements in the microarray clearly showed that most of the genes up-regulated by MSB contain the G-box in their promoter regions. Some interesting functions were represented among the individual up-regulated genes, such as glutathione S-transferases, transcription factors (including putative regulators of the G-box) and cytochrome P450s (Borges et al., 2009). In Figure 1 we propose a hypothetical model that summarizes the possible mode of action of MSB as priming agent in planta.

Another notable effect of MSB is its capacity to induce a reduction in insect growth rate (*unpublished*). Interestingly, a recent MSB application has been patented for controlling *Trioza erytreae* and *Diaphorina citri*, the psyllid vectors carrying the genus *Candidatus Liberibacter* that are bacterial causal agents of the most serious citrus disease known as Huanglongbing (HLB) (Borges-Rodríguez and Borges-Pérez, 2010).

Another effect of menadione on abiotic stresses is to induce tolerance to chilling stress in maize seedlings (Prasad et al., 1994). These authors suggested that exogenous application of menadione and H_2_O_2_ to the seedlings might induce a mild oxidative stress leading to chilling tolerance (Prasad et al., 1994). A very recently published work from our laboratory has focused on the MSB effect at the seed stage (Jiménez-Arias et al., forthcoming). Firstly, we found that soaking Arabidopsis seeds in 20 mM MSB induces salt tolerance by priming an early plant adaptation and proline accumulation. In addition, it was found that MSB primes the expression of key transcription factors such as Zat12, one of the key zinc-finger proteins encoded by a multi-gene family and involved in a ROS-dependent signaling network against abiotic stress (Mittler et al., 2011). Interestingly, it was also found that MSB leads to a hypomethylation state in the promoter region of genes involved in the biosynthesis (PYRROLINE-5-CARBOXYLATE SYNTHETASE 1, *P5CS1*) and degradation (EARLY RESPONSIVE TO DEHYDRATION 5, *ERD5*) of proline, demonstrating that one of the mechanisms underlying this early adaptation to salt stress is an epigenetic mark (*submitted for publication*).

### Conflict of interest statement

The authors declare that the research was conducted in the absence of any commercial or financial relationships that could be construed as a potential conflict of interest.
